# The Ideal Physician

**Published:** 1885-02

**Authors:** 


### The Ideal Physician.

Professor Gairdner thus describes him:

1. He must be careful and accurate, and at the same time a keen and quick observer of nature.

2. He must be able to connect his isolated observations of fact by rapid, and at the same time trustworthy, processes of reasoning.

3. He must, in dealing with emergencies, endeavor to have always what the Greeks (and Dr. John Brown after them) called *agchiouia*—nearness of the *nous*, *i. e.*, presence of mind.

4. He must, as a surgeon, have much deftness of manipulation—manual dexterity, as we call it; or perhaps still better, ambi-dexterity.

5. He must treasure in his memory, and be constantly increasing from day to day, large stores of various reading in his own and other languages, in order that not only past observations, but also the vast field of scientific progress in its relation to his art may be constantly before him, or at least freely accessible when wanted.

6. He must be able to write, at the very least, in his own language, with vigor, compactness, and lucidity.

7. He must have a soul above mere money-grubbing; must on no account degrade his profession into a trade; but must be, as far as is possible to human nature, the disinterested friend, the companion, the good genius, I had almost said, of all his patients.

8. For this reason, if no other, he must in every case have in him the distinctive essence of what is called a gentleman; and if his practice is, or is ever to be, among what are called the upper classes, he must be a gentleman, not only in principle but in detail; not necessarily what is vulgarly and falsely often styled a fine gentleman, but a gentleman in outward manner as much as in the inner spirit.

9. He must be a man endowed with a deep sense of moral responsibility, so as to beget confidence and unfailing trust in him on the part of his fellow-men. Responsibility, therefore, to them in the first instance; but underlying that, and sustaining it as surely as the root and the stem sustain the flower—a deeper and more latent responsibility to Him who is the source of all good, and, therefore, of all moral principle and moral responsibility whatever.

